# Emerging Functions of ICAM-1 in Macrophage Efferocytosis and Wound Healing

**DOI:** 10.33696/immunology.2.051

**Published:** 2020

**Authors:** Prarthana J. Dalal, Ronen Sumagin

**Affiliations:** Department of Pathology, Northwestern University Feinberg School of Medicine, Chicago, IL 60611, USA

**Keywords:** ICAM-1, Efferocytosis, Wound healing, Inflammation, Migration, Adhesion

## Introduction

ICAM-1 is a transmembrane, cell surface glycoprotein expressed by a variety of cells, but has been best-studied in vascular endothelium. Structurally, ICAM-1 is composed of five extracellular IgG- like domains to help facilitate cell-cell interactions and has a short cytoplasmic tail anchored to the cytoskeleton to facilitate intracellular signal transduction. ICAM-1 expressed by endothelial cells binds β2-integrins, CD11b/CD18 (Mac1), and CD11a/CD18 (LFA1) to mediate the adhesion of circulating leukocytes to the vessel wall and to facilitate transendothelial migration [1,2]. ICAM-1 can also regulate endothelial cell shape and vascular barrier function in response to inflammatory stimulation [3,4].

In addition to endothelial cells, ICAM-1 expression has been detected in vascular smooth muscle cells, pericytes, fibroblasts, keratinocytes, intestinal epithelial cells, and more recently in some subsets of immune cells [5–7]. For example, ICAM-1 expressed by T-cells helps deliver costimulatory signals for T-cell activation [8]. In dendritic cells, ICAM-1 helps bind T-cells and form immune synapses [9]. It has also been demonstrated that neutrophils can express ICAM-1 and increased neutrophilic ICAM-1 expression correlated with improved phagocytosis [10]. Macrophages were also found to express ICAM-1 [11], however, the role of ICAM-1 in macrophage function is an evolving area of investigation. This commentary specifically focuses on ICAM-1 in macrophages and its recently discovered role in facilitating efferocytosis.

## ICAM-1 and Efferocytosis

Efferocytosis is a specialized process for the clearance of apoptotic cells (ACs) by tissue macrophages. It is essential for maintaining tissue homeostasis and when impaired can lead to non-resolving pathologic inflammation and tissue injury. Effective efferocytosis involves recognition of AC-associated ligands by macrophages via specialized surface receptors, reorganization of the macrophage cytoskeleton during AC engulfment, as well as phagolysosome fusion within the macrophages to degrade the internalized ACs.

Macrophages express a number of efferocytotic receptors including the TAM family tyrosine kinases (Tyro3, Axl, and Mer) [12]. The active process of efferocytosis also results in cellular reprogramming of the macrophage and acquisition of pro-resolution phenotype. After AC ingestion, macrophages reduce proinflammatory cytokine production and concurrently increase the production of cytokines that dampen inflammation such as IL-10, transforming growth factor β (TGF-β), and prostaglandin E2 [13–15]. This signaling switch from pro-inflammatory state to pro-resolution state is key for mediating tissue repair. Therefore, defective efferocytosis both exacerbates inflammation due to accumulation of dead cells and cellular debris, as well as impairs the ability of macrophages to facilitate wound repair.

ICAM-1 expression has been previously shown to be induced in macrophages and to contribute to macrophage polarization [11,16–18]. Intriguingly, the way in which ICAM-1 regulates macrophage polarization appears to be context-dependent. For example, in the tumor microenvironment macrophage ICAM-1 was associated with a pro-inflammatory phenotype. However, in acute lung injury ICAM-1 was associated with a pro-resolution phenotype [17].

Given the important role of macrophage efferocytosis in injury resolution and the emerging role of ICAM-1 in macrophage effector function, our group recently examined macrophage ICAM-1 functionality in macrophages in the context of inflammatory bowel disease (IBD) [7]. IBD is a symptomatic, debilitating disease driven by injury to the intestinal epithelium and dysregulated immune responses [19,20]. As such, macrophages play an important role in both initiation and resolution of colon inflammation [21].

We found that ICAM-1 was indeed upregulated on inflammatory macrophages in the gut. Importantly, we identified a new function for ICAM-1 in mediating macrophage-AC binding and facilitating efferocytosis [7]. Whereas stimulation of bone-marrow derived macrophages by IL-4 to resemble the resident/pro-repair phenotype in culture did not induce ICAM-1 expression, macrophage differentiation towards the inflammatory phenotype by interferon γ (IFNγ) and lipopolysaccharide (LPS) stimulation robustly increased ICAM-1 expression. Similarly, in a model of murine colitis, inflammatory macrophages isolated from the colon showed increased ICAM-1 expression. This was additionally supported by data from human IBD patients. Tissue sections of colon with active IBD showed more macrophages that co-stained positive for ICAM- 1 compared to noninflamed colon sections. Macrophages lacking functional ICAM-1 receptor were unable to effectively perform efferocytosis *in vitro* and *in vivo* thus confirming the role of ICAM-1 in efferocytosis. Furthermore, antibody-mediated inhibition or knockdown of ICAM-1 both *in vitro* and *in vivo* significantly decreased the ability of macrophages to engulf and uptake ACs. We also established that ICAM-1 clustered at the site of the engulfed ACs mediating their binding to macrophages.

**Figure 1:**
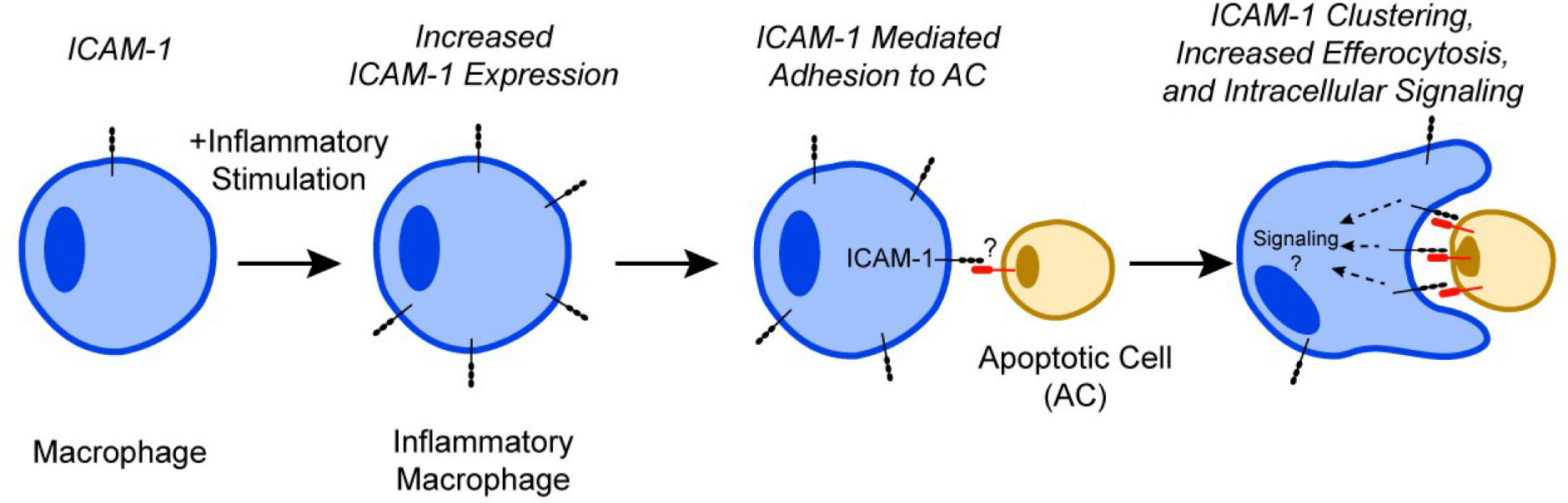
Schematic representation of the role of ICAM-1 in macrophage efferocytosis. ICAM-1 expression is induced in inflammatory macrophages. This mediates the binding of apoptotic cells (ACs) to promote uptake. Macrophage ICAM-1 clusters around ACs to facilitate increased efferocytosis and intracellular signaling.

These data demonstrate that ICAM-1 when induced in inflammatory macrophages plays an important role in binding of ACs during efferocytosis (summarized by the schematic, Figure 1).

These findings also raise additional important questions that should be considered in future investigation. For example, it is still unclear how ICAM-1 expression is regulated in macrophages. Increased iNOS and reactive oxygen species (ROS) have been suggested to promote ICAM-1 upregulation but given the diversity of macrophage phenotypes and their plasticity it remains to be seen whether there is a particular subset of inflammatory macrophages that undergo this change [18]. Similarly, given the variety of cells that macrophages can clear in various tissues, ICAM-1 ligands on ACs should be investigated in more detail. While in the case of immune cells, these interactions may be facilitated by ICAM-1 binding to β2-integrins, efferocytosis of epithelial cells or fibroblasts, which do not express β2-integrins will be mediated by other yet undefined ligands.

## ICAM-1 and Wound Healing

Wound healing is a complex, multi-step cascade of events that requires careful spatial and temporal synchronization of a variety of different cells and processes. Key hallmarks of the wound healing process include hemostasis, inflammation, cellular proliferation, and tissue remodeling [22]. ICAM-1 has been previously implicated in facilitating wound repair via several implied but not well-defined mechanisms. For example, skin wound healing in ICAM-1 KO mice was significantly delayed [23], a phenotype that was associated with reduced wound infiltration of neutrophils and macrophages and reduced granulation tissue formation [24]. These observations are consistent with the role of endothelial cell ICAM-1 in mediating leukocyte transendothelial migration and the well-recognized contributions of both cell types to initiation of inflammation, host defense and injury resolution.

Similar to skin, repair after colonic injury was also significantly delayed in the absence of functional ICAM-1. Following injury to the colonic mucosa, ICAM-1 expression was highly induced in colon epithelial cells, facilitating neutrophil apical retention and promoting epithelial cell proliferation and wound closure [5,25]. This work has clearly demonstrated that the impairment in mucosal healing under these conditions was driven by ICAM-1 expression by epithelial cells. However, our recent findings demonstrating ICAM-1 expression on macrophages suggest that this may also be involved to the observed phenotype. We found that macrophage ICAM-1 is essential for facilitating efferocytosis and without efferocytosis, the resolution of inflammation and restoration of tissue homeostasis cannot occur. Furthermore, efferocytosis drives macrophage reprogramming and polarization towards a pro-resolution phenotype and promotes crucial healing effector functions of macrophages. Particularly in the gut, where the majority of tissue resident macrophages are derived from circulating monocytes, ICAM-1 expressed by various cell types appears to coordinate several important aspects of wound healing including monocyte recruitment into the wounded tissue (endothelial ICAM-1), wound debridement and resolution of inflammation (macrophage ICAM-1) and wound reepithelialization (epithelial cell ICAM-1).

In addition to mediating cellular adhesion, ICAM-1 also serves as a signaling receptor. ICAM-1 engagement is associated with calcium signaling, Rho activation, Akt/β-catenin signaling, and is also coupled with cytoskeletal remodeling [3,25,26]. However, whether ICAM-1 signals in macrophages and whether this impacts efferocytosis remains unknown. This should be examined in future studies, as calcium influx, Rho activation, and cytoskeletal changes are all critical elements for AC engulfment and for macrophage effector function.

Finally, ICAM-1 has been previously considered as a therapeutic target by several clinical trials however, without apparent success. For example, ICAM-1 inhibition was tested in clinical trial to treat ischemic stroke [27]. Given the emerging pro-resolution and pro-healing functions of ICAM- 1, this is perhaps not surprising. Current emerging identification of its roles in wound healing in specific cell types provides a better opportunity for targeted therapy and rekindles interest in this important molecule. Improperly healing wounds in the context of many organs including skin, lung, and colon represent a significant healthcare burden. Thus, developing effective therapies to improve wound healing remains an active research focus and exploring the role of macrophage ICAM-1 can significantly improve our current understanding of this process.
